# First-episode mania after COVID-19: A case series in Iran

**DOI:** 10.3389/fpsyt.2023.1102450

**Published:** 2023-04-11

**Authors:** Mahdieh Saeidi, Tara Rezvankhah, Victor Pereira-Sanchez, Maryam Rafieian, Behnam Shariati, Soode Tajik Esmaeeli, Maziar Emamikhah, Kaveh Alavi, Amir Shabani, Shiva Soraya, Fatemeh Kashaninasab, Fatemeh Sadat Mirfazeli

**Affiliations:** ^1^Department of Psychiatry, Mental Health Research Center, School of Behavioral Sciences and Mental Health (Tehran Institute of Psychiatry), Iran University of Medical Sciences, Tehran, Iran; ^2^Grossman School of Medicine, New York University, New York, NY, United States

**Keywords:** COVID-19, mania, bipolar disorder, pandemic, first-episode mania

## Abstract

**Background:**

Increasing reports of manic episodes in patients during acute infection with COVID-19 have been documented since the pandemic began, including individuals without a previous personal or family history of bipolar disorder. As infections and autoimmunity have putative roles in bipolar disorder, we aimed to document the clinical presentations, associated stressors, family aggregation patterns, and brain imaging and electroencephalographic correlates with a series of patients with episodes of mania that emerged shortly after COVID-19 infections.

**Methods:**

We obtained all relevant clinical information from 12 patients whose first manic episode started within a month of COVID-19 infection and were treated at Rasool-e-Akram hospital and Iran psychiatric hospital, two tertiary medical centers in Tehran, Iran, in 2021.

**Results:**

Patients had a mean age of 44. The interval between the onset of symptoms of COVID and mania ranged between 0 and 28 days (mean: 16.25, median: 14 days); it was observed to be shorter in patients with a family history of mood disorders but not in those receiving corticosteroids. Alongside a descriptive overview of our sample, we provide detailed narrative descriptions of two of the cases for illustrative purposes and discuss our observations in the context of other cases reported elsewhere and the state-of-the-art regarding infectious diseases, COVID-19, and bipolar disorder as reported in previous literature.

**Conclusion:**

Our case series documents observational and naturalistic evidence from a dozen of cases of mania in the context of acute COVID-19, which, while limited, calls for analytical research of the phenomenon, and points at a family history of bipolar disorder and the use of corticosteroids as factors for particular focus.

## Background

While the COVID-19 pandemic remains a major global health challenge, research has advanced in understanding the highly heterogeneous and multisystem manifestations of the infection. Neuropsychiatric symptoms in patients with COVID-19, either as new-onset manifestations or exacerbations or relapses of preexisting conditions, have been repeatedly reported (1–4). Those are thought to be caused by the direct and indirect effects of the virus on the brain and by the psychosocial stressors brought up by the pandemic and containment measures (5, 6). Estimates of the prevalence of neurological and psychiatric symptoms in patients with COVID-19 are one-fifth and one-half, respectively (3). The neuropsychiatric manifestations of COVID-19 have been categorized into three distinct groups: olfactory symptoms; headache and limb force reduction; photophobia, hallucinations, mental state change, vision and speech problems, seizure, stroke, and ataxia (7). New-onset psychiatric illness has been identified in 5.8% of COVID-19 survivors within 14–90 days since the COVID-19 diagnosis (8).

Bipolar disorder (BD) is a spectrum of chronic, episodic, and highly disabling mood disorders with a lifetime prevalence of about 2.4% (9, 10) and is associated with poor health and reduced life expectancy due to psychiatric and general medical conditions, including suicide, respiratory and cardiovascular diseases, and cancer (11–13). A typical presentation of new-onset BD is first-episode mania, in which people without a previously known history of BD exhibit symptoms such as elevated or dysphoric mood, grandiosity, irritability, increased psychomotor activity, behavioral disinhibition, decreased need for sleep, distractibility, and, severe cases of, psychotic features such as delusions and hallucinations congruent with the mood (14). There have been increasing reports of first manic episodes in people within days after being infected by SARS-CoV-2 (15–22). While actual the causality, correlation, and pathophysiology of mania following COVID-19 are not yet understood, previous research associating BD with immune and inflammatory dysfunctions (23, 24) shows a way to study mania after COVID-19 as a possible result of direct and indirect brain effects of the virus.

Being first-episode mania, and consequently new-onset BD, a potential, rare yet very severe complication of COVID-19, it is paramount to identify affected individuals and characterize its manifestations and course. This study presents an original case series of 12 individuals presenting with first-episode mania within a month of being infected with COVID-19. It includes an overview of the series showing the most remarkable demographic and clinical details (symptoms, medical workup, treatment, etc.) of each case alongside two illustrative detailed cases. The series is accompanied by a compilation of other case reports previously published in international scientific literature to provide a more comprehensive and global understanding of the phenomenon and promote further discussion, data sharing, and research on the topic.

## Methods

This original case series aimed to document the clinical presentation, associated stressors, family aggregation patterns, and brain imaging and electroencephalographic correlates in hospitalized patients with post-COVID first-episode mania. Patients’ records were included in the series if they had their first manic episode within a month of COVID-19 infection and were excluded if patients were abusing substances or had any past medical history of a psychiatric or neurological disorder.

Twelve patients were included; they had attended Rasool-e-Akram hospital and Iran psychiatric hospital, two tertiary medical centers in Tehran, Iran, and were diagnosed with bipolar disorder type 1 (currently in the first episode of mania) by an experienced psychiatrist after a complete psychiatric assessment. COVID-19 had been diagnosed by an infectious disease specialist based on a positive RNA test and/or a spiral chest computerized tomography (CT) scan, these findings were reported by an experienced radiologist. Nine out of 12 patients had agreed to undergo brain magnetic resonance imaging (MRI) during hospitalization. Additional demographic and clinical data were collected by psychiatry residents through face-to-face interviews with the patients and their families during their hospitalization and through chart reviews of patients’ records. Information of interest included demographic details, past medical, psychiatric, substance, drug, family history, COVID-19 symptoms and diagnostic methods, treatment records, and paraclinical data. Descriptive statistics were obtained using SPSS v.16.0 software (SPSS Inc., Chicago, IL, United States).

Informed consent for inclusion in the reported series was obtained from all patients and their families, and the study follows the Helsinki Declaration (current version, 2013).

## Results

This case series included 12 patients with a mean age of 44 (median: 43, range: 32–69) including seven men and five women. Patients’ characteristics are presented in Tables 1-1 and 1-2. The interval between the onset of symptoms of COVID-19 and mania ranged between 0 and 28 days (mean: 16.25 days, median: 14 days). Five out of 12 patients had a family history of mood disorders (cases 2, 4, 6, 7, and 8; see tables for correspondence), and eight out of 12 patients had received corticosteroids as a treatment for COVID-19 (cases 1, 2, 3, 6, 7, 8, 11, and 12; see tables for correspondence). None of the patients had a previous personal psychiatric history and only one had past substance use (methamphetamine). Findings from neurological examination and imaging were unremarkable for all patients. There was no observed relevant difference between men and women regarding the long interval between the onset of symptoms of COVID-19 and mania onset (mean: 15.43 days for men, and 17.4 for women).

**Table 1-1:** Characteristics of patients with post-COVID mania in the case series.

Case	Age (years), sex (M, F)	Educational level	Interval between the onset of COVID-19 and the onset of psychiatric symptoms	Psychiatric symptoms	COVID-19 symptoms	Diagnostic method	COVID-19 treatment	PMH
1	40 M	Elementary school	4 weeks	irritability, decreased need for sleep, aggression, hallucination auditory, grandiosity delusion, thought racing, distractibility	fever, myalgia	positive COVID-19 PCR, significant changes in Spiral Chest CT scan	favipiravir, IV corticosteroid therapy	None
2	32 M	BSc	2 weeks	irritability, decreased need for sleep, aggression, hallucination auditory, grandiosity delusion, thought racing, distractibility	fever, sleep deprivation, myalgia, fatigue	positive COVID-19 PCR, significant changes in Spiral Chest CT scan	IV corticosteroid therapy, remdesivir	None
3	69\u00B0F	Elementary school	10 days	irritability, decreased need for sleep, aggression, hallucination auditory, grandiosity delusion, reference delusions, agitation	fever, myalgia	positive COVID-19 PCR	IV corticosteroid therapy	None
4	45\u00B0F	Elementary school	2 weeks	elevated mood, increased energy, decreased need for sleep, talkativeness, flight of ideas and thought racing, grandiosity delusion, overspending, increased religious goal directed activity	cough, hemoptysis, fatigue	Significant changes in Spiral Chest CT scan	–	None
5	62 M	BSc	4 weeks	Irritability, decreased need for sleep, talkativeness, overspending, aggressive behaviors, auditory hallucinations, grandiosity delusions, persecutory delusions	myalgia, fatigue, productive cough, dyspnea	positive COVID-19 PCR, significant changes in Spiral Chest CT scan	favipiravir	None
6	45 F	Diploma	2 weeks	irritability, talkativeness, aggression, elevated energy, suicidal ideation, visual and auditory hallucination, decreased need for sleep, persecutory delusions	fever, myalgia, shortness of breath	positive COVID-19 PCR, significant changes in Spiral Chest CT scan	IV corticosteroid therapy, remdesivir	None
7	36 M	PhD	2 weeks	irritability, talkativeness, aggression, elevated energy, increase in appetite, visual and auditory hallucination, decreased need for sleep	Myalgia, fatigue, productive cough, dyspnea	positive COVID-19 PCR, significant changes in Spiral Chest CT scan	IV corticosteroid therapy	None
8	44 M	BSc	10 days	irritability, decreased need for sleep, aggression (verbal and physical), talkativeness, increased energy, flight of ideas and thought racing, grandiosity delusions, visual and auditory hallucinations (religious content)	fever, cough, diarrhea, fatigue	positive COVID-19 PCR	IV corticosteroid therapy	None
9	45 M	Elementary school	2 weeks	Increased energy, irritability, decreased need for sleep, flight of ideas, grandiosity delusions	fever, myalgia, cough	Positive COVID-19 PCR	–	None
10	42 M	Diploma	concurrent	persecutory and grandiosity delusions, auditory hallucinations, irritability, increased energy, decreased need for sleep, flight of ideas, aggression	fever, fatigue	positive COVID-19 PCR	conservative therapy	IHD
11	37 F	Diploma	3 weeks	grandiosity delusions, auditory hallucinations, irritability, increased energy, decreased need for sleep, flight of ideas, aggression	fever, fatigue	positive COVID-19 PCR, significant changes in Spiral Chest CT scan	IV corticosteroid therapy, colchicine	intellectual disability
12	35 F	Diploma	4 weeks	irritability, flight of ideas, grandiosity delusions, visual hallucinations, decreased need for sleep, agitation not available/applicable aggression	fever, myalgia, fatigue	positive COVID-9 PCR	IV corticosteroid therapy, remdesivir	none

M, male; F, female; BSc, bachelor of science; PhD, doctor of philosophy; PMH, past medical history; PCR, polymerase chain reaction; CT scan, computed tomography; IHD, ischemic heart disease.

**Table 1-2:** Characteristics and imaging of patients with post-COVID mania in the current study.

Case	PPH	Psychiatric treatment	DH	FH	SH	Neuroimaging	EEG	ECT	CSF analysis
1	None	Na valproate 500 mg, HS, haloperidol 5 mg, TDS, biperiden 2 mg, HS, clonazepam 1 mg, HS	Negative	None	None	Normal	Unremarkable	3 sessions	Unremarkable
2	None	–	Negative	Bipolar disorder in first degree relatives	None	Normal	Unremarkable	Not available/applicable	Unremarkable
3	None	olanzapine 5 mg, TDS	Negative	None	None	Normal	Unremarkable	Not available/applicable	Unremarkable
4	None	Na valproate 500 mg BD, haloperidol 5 mg BD, quetiapine 100 mg, HS	Negative	Mood disorder in second degree relatives	None	Normal	Not available/applicable	Not available/applicable	Not available/applicable
5	None	Na valproate 500 mg, 1HS, risperidone 4 mg, 1HS, clonazepam 1 mg, HS	Negative	None	None	Mild senile atrophic changes, moderate small hypersignal lesions centrum semiovale, frontoparietal periventricular white matter basal ganglia, external capsules are seen (that could be due to small vessel ischemic changes), inflammatory changes in paranasal sinuses are seen, mild cistern magna widening is seen.	Not available/applicable	Not available/applicable	Not available/applicable
6	None	tab olanzapine 5 mg, D, Na valproate 500 mg, BD	Negative	Unipolar depression in first degree relatives	None	Normal	Unremarkable	Not available/applicable	Unremarkable
7	None	olanzapine 5 mg, 2 HS	Negative	Mood disorder in first degree relatives	None	Normal	Not available/applicable	Not available/applicable	Unremarkable
8	None	Na valproate500mg, TDS, risperidone 4 mg, 1.5HS	Negative	Mood disorder in first degree relatives	None	–	Not available/applicable	Not available/applicable	Not available/applicable
9	None	–	Negative	None	None	–	Not available/applicable	Not available/applicable	Not available/applicable
10	Substance (methamphetamine) induced psychotic disorder	olanzapine5mg, TDS, Na valproate 500 mg, TDS	Negative	None	methamphetamine	Normal	Not available/applicable	Not available/applicable	Not available/applicable
11	None	olanzapine5mg, BD, Na valproate 500 mg, BD	Negative	None	None	Normal	Abnormal due to diffuse slowing (tetha, delta waves) and focal poly sharp waves	Not available/applicable	Unremarkable
12	None	Na valproate500mg, TDS, quetiapine 100 mg, TDS, biperiden 2 mg, BD, perphenazine 8 mg, TDS	Negative	None	None	–	Not available/applicable	Not available/applicable	Not available/applicable

PPH-past psychiatry history, DH-drug history, FH-family history, SH-substance history, EEG-electroencephalogram, ECT-electroconvulsive therapy, CSF-cerebrospinal fluid, HS-hora somni/at night, BD-bis in die/twice a day, TDS-ter die sumendus/three times a day, D-once a day.

Patients with a family history of mood disorders had an observed shorter average interval (mean: 13.2, median: 14 days) compared to those with a negative family history (mean: 18.43, median: 21 days). The median interval between COVID-19 symptoms and mania onset was similar in patients with and without corticosteroid treatment and in those with a mood disorder family history (the median for all three groups was 14 days), while individuals without a mood disorder family history had a longer median interval of 21 days.

The Mann–Whitney *U* test was chosen from the nonparametric tests. There was no significant relationship between the family history of mood disorder and the distance between the onset of mania and COVID-19 (*p* value = 0.722), as well as the use or non-use of corticosteroids. There was no significant relationship between the time interval between the onset of mania and COVID-19 (*p* value = 0.308).

### Illustrative case 1

A 40-year-old man (case #1; see tables for correspondence), with no significant medical, psychiatric, family, or substance use history, was brought to the psychiatric emergency department. His companion reported an abnormal body movement (pseudo seizure) while he was awake and that he became aggressive and sexually disinhibited. He had exposed himself in front of others and said inappropriate sexual content. Based on the family member’s report, his sense of orientation was intact. Weeks earlier he had had fever and myalgia and had been assessed by an infectious disease specialist; the diagnosis of COVID-19 was supported by the polymerase chain reaction (PCR) and spiral chest CT scan. He was treated with favipiravir and intravenous corticosteroid therapy. While the symptoms of COVID-19 were improving, new-onset psychiatric symptoms emerged with an acute and progressive course: irritability, decreased need for sleep, thought racing, distractibility, aggression, auditory hallucinations (hearing God’s voice telling him about his ‘superpower’ and intelligence), and grandiosity delusions (being the ‘special servant of God, being able to read people’s minds and actions with closed eyes, having a great power to change the whole world). After admission to the hospital, a thorough physical and neurological workup was obtained, including brain CT and MRI, electroencephalogram (EEG), lumbar puncture, and urine toxicology, and the findings were unremarkable. Due to the unreliable history of a possible seizure just before hospitalization, neurologists started phenytoin 100 mg intravenous, three times a day, and after no evidence of a seizure, neurologists changed it to capsule phenytoin 100 mg three times a day and then tapered it to discontinuation during hospitalization.

His symptoms were severe and he was aggressive; he would remove his intravenous line and fight with the staff despite receiving several haloperidol injections alongside oral medication (sodium valproate 1,500 mg/day, haloperidol 5 mg three times a day, biperiden 4 mg/day, and olanzapine 5 mg per night). He, therefore, received three sessions of bilateral electroconvulsive therapy (ECT) and his symptoms improved significantly; he was eventually discharged in a state of clinical remission after 22 days of hospitalization with the following medications: sodium valproate 500 mg every night, haloperidol 5 mg three times a day, biperiden 2 mg every night, and clonazepam 1 mg every night.

### Illustrative case 2

A 32-year-old man (case #2; see tables for correspondence), with no previous history of psychiatric disorders, presented at the hospital emergency department with mood symptoms. The patient was irritable, restless, and aggressive. He had a decreased need for sleep, the pressure of speech, and paranoid ideations about his neighbors (reporting that they were watching him through the window and wanted to ruin his reputation). He also had been presenting an unusually inflated self-esteem and sense of importance in the previous days, and he reported feeling like his ‘brain was racing’. The patient had also been overspending money, with financial and legal consequences. According to the patient’s wife, these acute symptoms had emerged in the context of insomnia due to myalgia and other symptoms associated with COVID-19. The patient had a fever, lethargy, weakness, severe myalgia, and lung damage 2 weeks before going to the psychiatric emergency department. He had been treated with intravenous injections of corticosteroids and remdesivir, with symptomatic physical improvement and psychiatric worsening. He had no significant past use of tobacco or alcohol. His mother had a history of manic episodes with the diagnosis of bipolar disorder.

Upon assessment, findings from his physical and neurological examination were unremarkable, and his mental status exam recorded a pressure of speech, elevated mood, and grandiosity delusions perseverative in religious content. The grandiosity delusion consisted of a conviction of possessing great power and knowledge and having “the mission of saving human beings.” The patient also had auditory hallucinations, reportedly hearing “the voice of God” and showing hallucinatory behaviors such as self-talking and externally unmotivated spontaneous laughing.

After the patient was admitted to the psychiatric ward, a lumbar puncture was performed for diagnostic measures, in which the analysis of cerebrospinal fluid was reported to be normal (colorless, white blood count of 0, red blood count of 450, bacteria not seen). Both patient’s brain MRI and EEG have lacked significant abnormalities. The patient was diagnosed with BD type I, a current episode of severe mania with psychotic features according to the Diagnostic and Statistical Manual of Mental Disorders, fifth edition (DSM-5) and received pharmacotherapy (haloperidol 15 mg per day, sodium valproate 2,000 mg per day, biperiden 5 mg per day, clonazepam 1 mg per day, and quetiapine 50 mg per day). The patient was discharged after 27 days of hospitalization with a euthymic mood and without any psychotic symptoms. In the first 3 months of follow-up, the patient remained asymptomatic and medicated (haloperidol 7.5 mg per day, sodium valproate 1,500 mg per day, and biperiden 3 mg per day).

#### Cases reported In previous literature

We searched and reviewed other case reports previously published in international scientific literature to provide a global context of the phenomenon. The most relevant information to compare with our samples is compiled in Table 2.

**Table 2:** A review of previously studied post-COVID manic patients.

Author	Age (years), sex, country	COVID—Mania interval	PI	PPH	PMH	FH	COVID symptoms	Steroids	Mania medications	Brain imaging
Mawhinney et al. (19)	41, male, United Kingdom	11 days	Elevated mood, agitation, disinhibition, racing thoughts, grandiosity delusions, persecutory delusions	A history of paranoid features after cannabis use 16 years ago	Congenital nystagmus	Postpartum psychosis and B1D in sister	Dry cough, fever, headache	None	olanzapine 10 mg daily, clonazepam 1 mg BD	MRI: Hyperintense signal in the splenium of the corpus callosum + decreased diffusion coefficient
Sen et al. (21)	33, female, Turkey	Concurrent	Irritability, decreased need for sleep, dysphoric mood, derailment of thoughts, persecutory, mystic and infidelity delusions	None	None	None	Sore throat and fever	None	olanzapine 20 mg/day	No MRI
Shaojia Lu et al. (17)	51, male, China	17 days	Irritability, decreased need for sleep, increased energy, talkativeness, grandiosity delusions	None	None	None	Pharyngalgia, fever, shortness of breath	Methylprednisolone	olanzapine 10 mg/day	MRI: Small ischemic lesions at Basal ganglia and semiovale center
Noone et al. (20)	49, male, USA	3 weeks	Dysphoric mood, grandiosity delusion, auditory hallucination, disorientation	None	HTN, HLP, DM2	None	Unknown	None	olanzapine 2.5 mg/day and then quetiapine up to 150 mg/day	Unremarkable MRI
Noone et al. (20)	34, female, USA	Concurrent	Irritable mood, agitation, decreased need for sleep, pressure of speech, distractibility, disorganized behavior, persecutory delusion	None	None	None	Unknown	None	Risperidone 1 mg BD	MRI: Nonspecific foci of T2 hyperintense signal abnormality in the right parietal subcortical white matter
Devasia et al. (25)	53, male, India	8–10 days	Irritability, decreased need for sleep, pressure of speech, grandiosity delusion, persecutory delusion, flight of ideas	None	None	B1D in brother	Sore throat, fever, myalgia, headache, anosmia	None	Risperidone 2 mg/day + Carbamazepine 400 mg/day	MRI: Small vessel ischemic changes
Shanmugam et al. (22)	52, male, United Kingdom	2 weeks	Elevated mood, overspending, aggression, excessive toilet cleaning, pressure of speech, disinhibition, grandiose ideas	None	HTN	None	High fever, diarrhea, mild headache, dry cough, anosmia	None	High dose Olanzapine + Sodium valproate	Unremarkable MRI
Reinfeld et al. (26)	Early 50s, male, India	Concurrent	Irritability, decreased need for sleep, pressure of speech, paranoid delusion, Sometimes staring with features of excited catatonic, fluctuating orientation	None	None	None	Low-grade fever, tachycardia, lung involvement	None	Electroconvulsive therapy	No MRI
Varsak et al. (27)	64, female, Turkey	12 days	Irritable mood, euphoria, agitation, increased energy, talkativeness, grandiosity delusion	None	None	None	Fever, myalgia, headache, diarrhea, taste and smell disorder	Methylprednisolone	olanzapine 20 mg/day uclopenthixol decanoate every 2 weeks (2 doses)	Unremarkable brain CT Scan
Uzun et al. (28)	16, male, Turkey	10 days after the end of infection	Irritability, euphoria, increased energy, and decreased sleep, talkativeness	None	Cerebral palsy	None	Mild symptoms	None	risperidone 3 mg/day, lithium 1,200 mg/day	No MRI

PI, present illness; PPH, past psychiatric history; PMH, past medical history; FH, family history; B1D, bipolar disorder type 1; MRI, magnetic resonance imaging; BD, bis in die/twice a day; HTN, hypertension; HLP, hyperlipidemia; DM2, diabetes mellitus type2.

## Discussion

Our case series includes 12 patients with similar presentations of manic episodes within a month of being infected with COVID-19, which bears similarities with other cases reported in the international scientific literature. While inferences and generalizations cannot be made through the clinical description of a local, small sample, and an actual association between COVID-19 and mania cannot be confirmed beyond temporal concurrence, our observations provide valuable insights for the formulation of hypotheses and the design of future systematic observations and inferential research.

The emergence of psychopathology during a major infectious disease outbreak such as COVID-19 manifested as new-onset, relapse, or exacerbation is understood as possibly associated with direct and indirect neurotropic effects of pathogens and the immune response they trigger, as well as with the psychological stress produced by the trauma and social and economic impacts of the outbreak and containment measures (29–32). COVID-19 is a particular case of a global pandemic with a massive social impact caused by a virus with neurotropic potential (31, 33), which makes it plausible to trigger manic relapses in patients with BD and new-onset episodes in predisposed individuals, especially those with a family history of BD. In these regards, insights from scientific literature related to the neuropsychiatric effects of viral diseases and the mental health impact of disasters are relevant to contextualize the discussion of our case series.

Regarding potential neurotropic pathways for COVID-19 to trigger manic episodes, previous research has associated several viral infections with BD, including Epstein–Barr virus (EBV), cytomegalovirus (CMV), herpes simplex virus (HSV), hepatitis B virus (HBV), and hepatitis C virus (HCV) (34–38). It has been hypothesized that an immune/inflammatory mechanism could induce changes in neurotransmitters at the limbic network (39), and several studies have found associations between BD and inflammation (34, 35, 40, 41). One study found increased C reactive protein during acute episodes of BD (42); another study reported a significant difference in peripheral blood lymphocytes which correlated with each phase of BD type 2, suggesting that cytotoxic T lymphocytes would migrate from blood to the brain in acute episodes of BD (43). As T cells are known for their defensive role against intercellular pathogens, especially viruses (44), these data support an underlying immune mechanism for BD and could help clarify the emergence of mania during acute infection by SARS-CoV-2. There has been a brain organoid study in which both indirect neuroinflammation and direct neuronal invasion of the virus have played a role in the neuropsychiatric manifestations of COVID-19 (33).

In terms of psychosocial stressors, Matsumoto et al. documented manic episodes in patients with BD in remission during the Great East Japan Earthquake and subsequent Fukushima nuclear disaster; notably, those BD relapses consisted more in manic rather than depressive episodes and were more observed in women than men (45). The COVID-19 pandemic unleashed a wide array of acute and chronic stressors including fear of being infected or infecting others, restrictive nationwide lockdown policies facilitating social isolation, domestic violence, boredom, inappropriate sleep hygiene, financial strain, trauma, and grief associated with the loss of loved ones, misinformation, insufficient social support of vulnerable populations, including the elderly with cognitive decline, social stigma, and limited access to care (46–48).

Patients with a family history of BD in our series had an earlier mania onset while they had normal or unremarkable paraclinical data. While speculative, it might be possible that psychosocial stress and treatment with corticosteroids would be the main triggers of acute mania in those with a family history, while neurotropic damage by the virus would have a more prominent role in cases without such evident genetic vulnerability. However, in previous case reports some patients with a family history presented abnormal neuroimaging findings (19, 25).

Corticosteroid therapy is one of the major components of the treatment of many cases of severe COVID-19, and it poses a well-known potential to induce acute neuropsychiatric conditions, including typically, mania, which gives them a possible relevant role in some of our cases. Corticosteroid-induced psychiatric symptoms are dose-dependent and tend to occur during the first weeks after initiation (49, 50). The main underlying mechanisms are unclear but there is evidence of disturbances in glucocorticoid stimulation and mineralocorticoid receptor stimulation which cause glutamate-induced neuronal toxicity (51). To note, there seemed to be no relevant difference in the interval between COVID-19 and mania onset in patients receiving those treatments in our series as compared to those not receiving them.

It is essential to note the main limitations of the evidence presented in this study. First of all, we had a small sample size and missing data, including clinical and imaging for some of our patients. Second, other than COVID-19, corticosteroids, and a family history of mood disorders, there could be many other factors playing a role in the emergence of mania in our patients that we have not contemplated or our data are not able to capture. Third, there was no follow-up for all patients after discharge. Finally, the nature of our study cannot present inferential evidence about the putative role of COVID-19 as a cause or a risk factor in the emergence of the first episode of mania. There is a great need for further research regarding the risk and protective factors for patients to present a first manic episode after COVID-19 infection, potentially identifying the high-risk groups that need closer follow-up.

While the pathophysiology of BD, involving complex interactions of genetic, epigenetic, and environmental predisposing and triggering factors, remains unclear (34, 39), future systematic research of the phenomenon of mania in the context of COVID-19 infection could provide valuable insights to inform the understanding and management of this disorder in contexts beyond COVID-19.

## Conclusion

Our series provides observational, naturalistic evidence regarding the phenomenon of first-episode mania after an acute COVID-19 infection, and questions whether a family history of bipolar disorder and the use of corticosteroids could be triggering factors. Clinicians treating patients with COVID-19 should be aware of the possibility of the emergence of mania and other psychiatric disorders in the course of infection and its treatment, considering the putative roles of inflammation and pharmacological iatrogenic and trying to identify patients with potentially higher risk for those conditions.

## Data availability statement

The raw data supporting the conclusions of this article will be made available by the authors, without undue reservation.

## Ethics statement

The studies involving human participants were reviewed and approved by Ethics Research Committee of the Iran University of Medical Sciences with unique number IR.IUMS.REC.1399.080 and protocol number 17630. The patients/participants provided their written informed consent to participate in this study. Written informed consent was obtained from the individual(s) for the publication of any potentially identifiable images or data included in this article.

## Author contributions

MS and TR: contribution in the design, acquisition of data, drafting, approval and agreement to be accountable for all aspects of the work. VP-S: contribution in the conception of data, interpretation of data, revising, approval and agreement to be accountable for all aspects of the work. MR, BS, SE, AS, and KA: contribution in the acquisition of data, drafting, approval and agreement to be accountable for all aspects of the work. BS and ME: contribution in the acquisition of data, revising, approval and agreement to be accountable for all aspects of the work. FM: contribution in the design and conception, acquisition of data, interpretation, revising. All authors contributed to the article and approved the submitted version.

## Conflict of interest

The authors declare that the research was conducted in the absence of any commercial or financial relationships that could be construed as a potential conflict of interest.The handling editor RF declared past collaborations with one of the authors VP-S.

## Publisher’s note

All claims expressed in this article are solely those of the authors and do not necessarily represent those of their affiliated organizations, or those of the publisher, the editors and the reviewers. Any product that may be evaluated in this article, or claim that may be made by its manufacturer, is not guaranteed or endorsed by the publisher.

## Abbreviations

COVID-19: Coronavirus disease 2019
BD: bipolar disorder
RNA: ribonucleic Acid
PCR: polymerase chain reaction
CT scan: computerized tomography scan
MRI: magnetic resonance imaging
EEG: electroencephalogram
ECT: electroconvulsive therapy
DSM-5: diagnostic and statistical manual of mental disorders, fifth edition
EBV: Epstein–Barr virus
CMV: cytomegalovirus
HSV: Herpes simplex virus
HBV: Hepatitis B virus
HCV: Hepatitis C virus
